# The anti-angiogenic effect and novel mechanisms of action of Combretastatin A-4

**DOI:** 10.1038/srep28139

**Published:** 2016-06-24

**Authors:** Min Su, Jingjia Huang, Suyou Liu, Yuhang Xiao, Xiyuan Qin, Jia Liu, Chaoqiong Pi, Tiao Luo, Jijia Li, Xianghui Chen, Zhiyong Luo

**Affiliations:** 1 Molecular Biology Research Center, State Key Laboratory of Medical Genetics, School of Life Sciences, Central South University, Changsha 410078, China; 2 Department of Medicinal Chemistry, School of Pharmaceutical sciences, Central South University, Changsha 410006, China

## Abstract

Combretastatin A-4 (CA4) is the lead compound of a relatively new class of vascular disrupting agents that target existing tumor blood vessels. Recent studies showed the CA4 might inhibit angiogenesis. However, the underlying molecular mechanisms by which CA4 exerts its anti-angiogenic effects are not fully understood. In this study, we revealed that CA4 inhibited vascular endothelial growth factor (VEGF)-induced proliferation, migration and capillary-like tube formation of human umbilical vascular endothelial cells (HUVECs). In *in vivo* assay, CA4 suppressed neovascularization in chicken chorioallantoic membrane (CAM) model and decreased the microvessel density in tumor tissues of a breast cancer MCF-7 xenograft mouse model. In addition, CA4 decreased the expression level and secretion of VEGF both in MCF-7 cells and HUVECs under hypoxia, as well as the activation of VEGFR-2 and its downstream signaling mediators following VEGF stimulation in HUVECs. Moreover, VEGF and VEGFR-2 expression in tumor tissues of the mouse xenograft model were down-regulated following CA4 treatment. Taken together, results from the current work provide clear evidence that CA4 functions in endothelial cell system to inhibit angiogenesis, at least in part, by attenuating VEGF/VEGFR-2 signaling pathway.

Growth and metastasis of solid tumors depend on angiogenesis, the formation of new blood vessels by endothelial cells from existing microvessel^1^. These new blood vessels grow into the tumor and provide the necessary oxygen, nutrients and growth factors for tumor progression. The inhibition of angiogenesis is explored as therapeutic prospect to treat cancer^2^,^3^. There are many steps which are critical for angiogenesis and blood capillary formation including endothelial cell survival, proliferation, migration, organization and remodeling into capillary-like structure^4^. It is logical that the anti-angiogenic efficacy of a compound will be directly correlated with a number of these steps being targeted by the agent.

Tumor-induced angiogenesis is mainly sustained by the production of angiogenic factors secreted from tumor cells. There are about 30 known endogenous pro-angiogenic factors participating in angiogenesis, among which, the vascular endothelial growth factor (VEGF) is the most potent mediator of angiogenesis^5^,^6^. It is now well indicated that VEGF stimulates numerous steps within the tumor angiogenesis, including endothelial cells survival, proliferation, migration, and invasion^7^. The *in vivo* angiogenic response to VEGF is mainly mediated via activation of VEGF receptor 2 (VEGFR-2)^8^. After binding with VEGF, VEGFR-2 on the surface of endothelial cell would undergo phosphorylation. The downstream signal pathways of VEGFR-2, which sequentially promotes angiogenesis, will be activated^9^,^10^. Therefore, interruption of VEGF/VEGFR-2 signaling pathway is considered to be a promising strategy interfering with solid tumor angiogenesis and tumor growth^11^.

It has been demonstrated consistently that microtuble-targeted drugs, such as taxanes, vinca alkaloids or combretastatins, can exhibit the most effective anti-angiogenic activities^12^. Combretastatin A4 (CA4) is a lead vascular disrupting compound undergoing clinical research^13^. It is a tubulin binding agent that binds to the colchicine binding site^14^,^15^. Much interest has recently been generated in the anti-angiogenic effects of CA4. Some previous studies reported that CA4 induced regression of tumor neovessels through interference with vascular endothelial-cadherin signaling^16^. In a recent study, Ren *et al*. reported that the anti-angiogenic effect of CA4 was associated with the Raf-MEK-ERK and Rho/Rho kinase signalling pathways^17^. Nevertheless, the molecular mechanism behind the anti-angiogenic effects of CA4 remain poorly understood. As VEGF/VEGFR signaling pathway is increasingly recognized as a key regulator during tumor angiogenesis, we here investigates the effect of CA4 on VEGF/VEGFR signaling.

In the present study, we showed that CA4 inhibited VEGF-induced proliferation, migration and capillary-like tube formation of endothelial cell, which are all important angiogenesis steps. CA4 also significantly suppressed VEGF expression and secretion both in MCF-7 cells and HUVECs, as well as VEGF-induced activation of VEGFR-2 and its downstream signaling transduction mediators in endothelial cell. In addition, treatment with CA4 decreased the protein expression of VEGF and VEGFR-2 in tumors of a breast xenograft mouse model. Taken together, our finding provides new insights into the mechanisms of tumor angiogenesis as well as tumor growth inhibited by CA4.

## Results

### CA4 inhibited proliferation of human cancer cells

**Figure 1:**
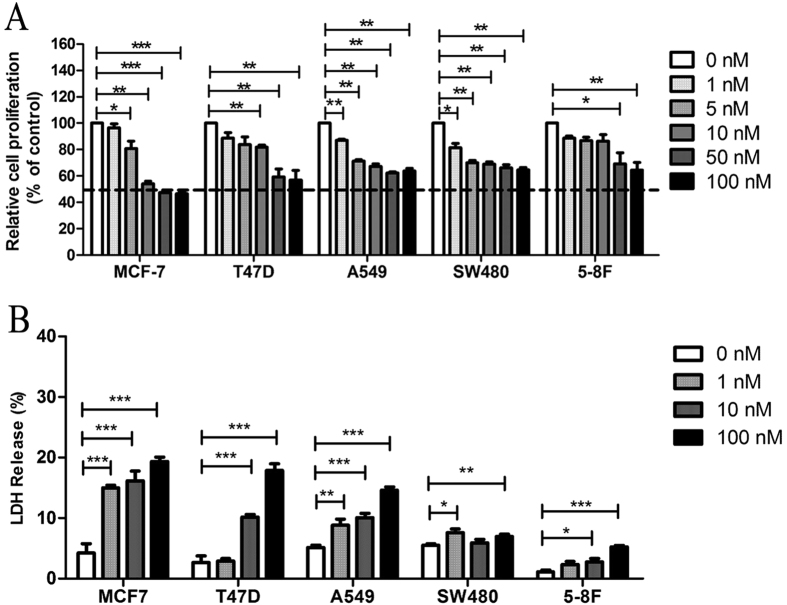
CA4 inhibited proliferation and induced cytotoxicity in human cancer cells. Human cancer cells proliferation was determined by MTT assay after being treated with CA4 for 48 h (**A**). Cytotoxicity of CA4 in human cancer cells was determined by LDH assay after being treated with CA4 fro 48 h (**B**). All data were represented as means ± SD, n = 3, *P < 0.05 versus control, **P < 0.01 versus control, ***P < 0.001 versus control.

The MTT assay results showed that treatment with CA4 led to dose-dependent inhibition of proliferation in human breast cancer cell line MCF-7 and T47D, human lung adenocarcinoma cell line A549, human colon cancer cell line SW480 and nasopharyngeal cancer cell line 5-8F (Fig. 1A). The IC_50_ value of CA4 in MCF-7 cells was between 10 nM and 50 nM. However, the maximal inhibitory effects of CA4 at 100 nM on other cells were less than 50%. This suggests that MCF-7 is the most sensitive to the effects of CA-4 among the tested cancer cells.

To validate whether or not CA4 would result in toxicity effects on human cancer cells, LDH cytotoxicity assay was carried out. As shown in Fig. 1B, treatment with CA4 in a concentration of 100 nM increased LDH release in all cell lines. Consistent with the proliferation inhibition results, MCF-7 cells were the most sensitive to CA4. Treatment with CA4 at concentrations of 1, 10 and 100 nM for 48 hours resulted in 10.8%, 11.9% and 15.1% addition of LDH release for MCF-7 cells, respectively.

### CA-4 inhibited proliferation of human endothelial cells

**Figure 2:**
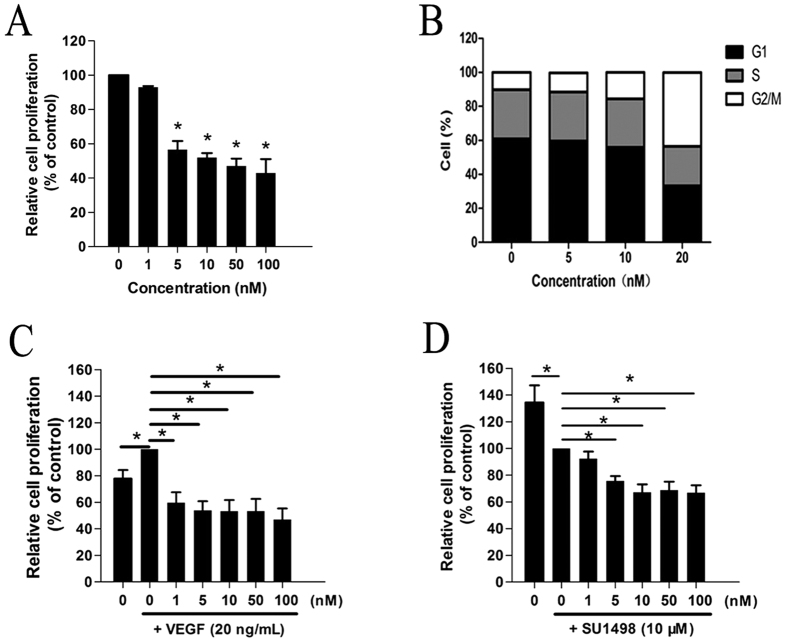
CA4 inhibited proliferation of human endothelial cells. Cell proliferation was determined by MTT assay after being treated with CA4 for 48 h (**A**). Analysis of cell cycle in HUVECs after being treated with CA4 for 24 h (**B**). Cell proliferation was determined by MTT assay after being treated with CA4 for 48 h in the presence of VEGF (20 ng/mL) (**C**). Cell proliferation was determined by MTT assay after being treated with CA4 for 48 h in the presence of SUl498 (10 μM) (**D**). All data were represented as means ± SD, n = 3, *P < 0.05 versus control, **P < 0.01 versus control, ***P < 0.001 versus control.

Endothelial cells proliferation plays an important role in the process of angiogenesis from preexisting vessels. To investigate the anti-angiogenic activity of CA4 *in vitro*, we first evaluated its inhibitory effects on the proliferation of HUVECs. As shown in Fig. 2A, we found that CA4 inhibited HUVECs proliferation in a dose-dependent manner. Given the correlation between cell cycle arrest and cell proliferation, we also examined the effect of CA4 on cell cycle progression of HUVECs by flow cytometry. Our data showed that CA4 dramatically induced cell cycle arrest at the G2/M phase (Fig. 2B).

To evaluate the role of VEGF/VEGFR-2 signaling in endothelial cell proliferation, HUVECs were treated with VEGF and VEGFR-2 specific inhibitor SU1498. As shown in Fig. 2C,D, the cell proliferation was increased up to 1.3-fold under VEGF (20 ng/mL) stimulation and decreased by 27% after SU1498 (10 μM) treatment. The results also showed that CA4 potently inhibited VEGF-induced proliferation of endothelial cell.

### CA4 blocked VEGF-induced endothelial cell migration

**Figure 3:**
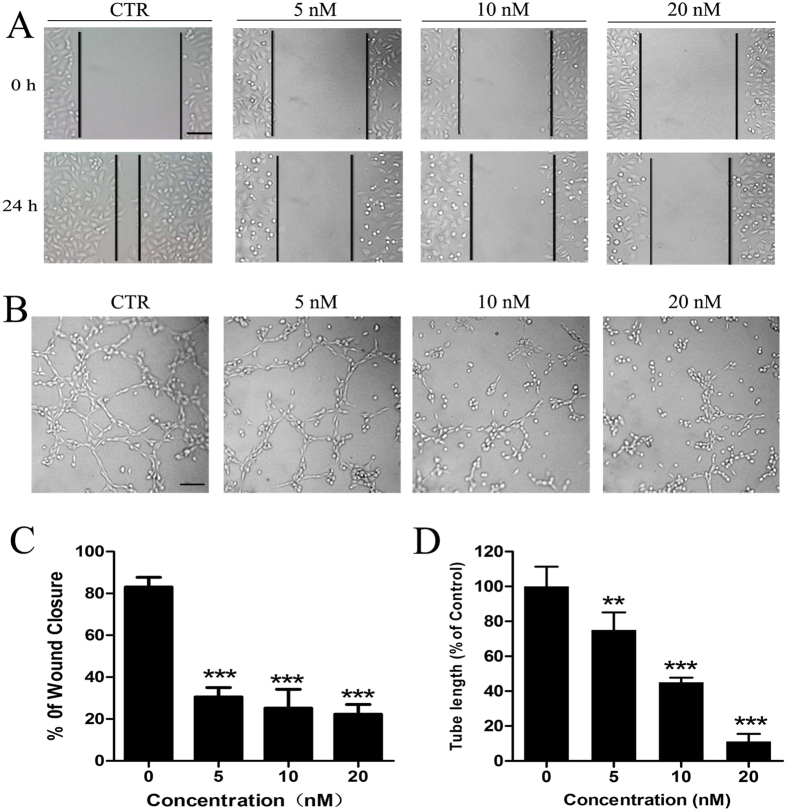
CA4 inhibited angiogenesis *in vitro*. Representative images of scratch wound-healing migration in HUVECs treated with CA4 for 24 h under VEGF stimulation (10×, bar = 20 μm) (**A**). Representative images of tube formation after being treated with CA4 for 2 h following VEGF stimulation (10×, bar = 20 μm) (**B**). Quantitative data of scratch wound-healing migration in HUVECs treated with CA4 for 24 h under VEGF stimulation (**C**). Quantitative data of tube formation after treated with CA4 for 2 h following VEGF stimulation (**D**). All data were represented as means ± SD, n = 3, *P < 0.05 versus control, **P < 0.01 versus control, ***P < 0.001 versus control.

Migration of vascular endothelial cells plays an essential role in the angiogenic process. Therefore, we used a wound-scratching migration assay to investigate the effects of CA4 on VEGF-induced endothelial cell migration. According to Fig. 3A,C, 24 hours after wounding, VEGF–treated endothelial cells could be seen to migrate into the denuded area. We also observed that treatment with CA4 dramatically inhibited the migration of HUVEC in a dose-dependent manner. These results suggest that VEGF-stimulated endothelial cell migration and angiogenesis might be inhibited specifically by CA4.

### CA4 inhibited VEGF-induced endothelial cell capillary-like tube formation

At later stages of angiogenesis, endothelial cells rearrange themselves into a tube to form a small blood vessel. We further investigated the effects of CA4 on VEGF-induced endothelial cell capillary-like structure formation using HUVECs grown on Matrigel *in vitro*. As shown in Fig. 3B,D, HUVECs incubated on Matrigel for 2 h formed an extensive and enclosed network of capillary-like tubes under the stimulation of VEGF. However, in the presence of CA4, the tube formation was interrupted in a concentration-dependent manner. Collectively, these results strongly suggest that CA4 specifically regulates VEGF-induced angiogenesis *in vitro*.

### CA4 blocked angiogenesis from aortic rings

**Figure 4:**
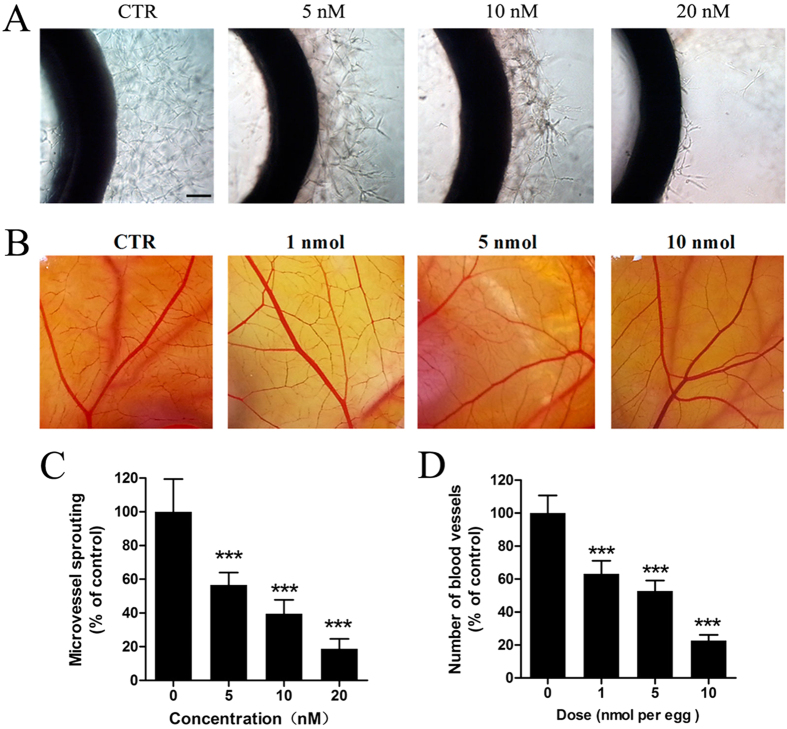
CA4 inhibited angiogenesis *ex vivo* and *in vivo*. Representative images of rat aorta sections after vehicle or CA4 treated for 6 d (10×, bar = 20 μm) (**A**). Representative images of chick embryonic CAM after treated with CA4 for 48 h (**B**). Quantitative data of rat aorta sections after treated with CA4 for 6 d (**C**). Quantitative data of chick embryonic CAM after treated with CA4 for 48 h (**D**). All data were represented as means ± SD, n = 5, *P < 0.05 versus control, **P < 0.01 versus control, ***P < 0.001 versus control.

When a section of aorta ring is embedded in Matrigel and cultured, microvessels can be observed growing out of the aorta ring and this mimics several stages in angiogenesis^18^. As a result, we evaluated the effects of CA4 on the capillary sprout outgrowth from rat aortic rings. As shown in Fig. 4A,C, a large number of microvessel structures were formed from rat aortic rings in the control group, while treatment with 5 nM CA4 notably suppressed microvessels formation and 20 nM CA4 completely blocked microvessels sprouting. These results suggest that CA4 blocked angiogenesis *ex vivo*.

### CA4 inhibited angiogenesis in CAM model

We also evaluated *in vivo* anti-angiogenic activity of CA4 by using the CAM assay. As illustrated in Fig. 4B,D, treatment with CA4 for 48 h caused a dose-dependent decrease in the branching of new capillaries from the exiting basal vessels compared with the control group. Quantitative analysis revealed that CA4 in doses of 1, 5 and 10 nmol/egg reduced blood vessels by 26.8%, 47.2% and 77.3%, respectively. These results suggest that CA4 inhibited angiogenesis *in vivo*.

### CA4 reduced VEGF expression

**Figure 5:**
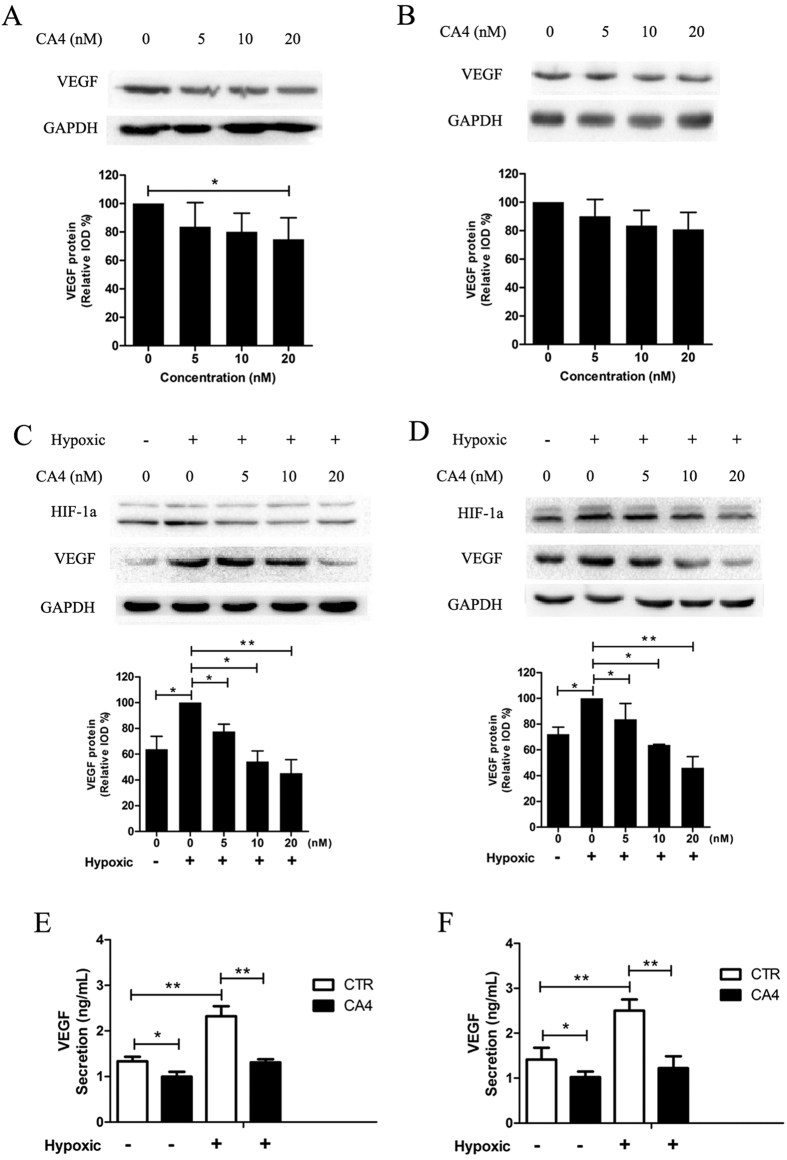
CA4 suppressed VEGF expression. A representative result (up) and histogram (down) of Western blot presents the levels of VEGF in MCF-7 cells (**A**) and HUVECs (**B**) treated with CA4 for 24 h at normoxia. A representative result (up) and histogram (down) of Western blot presents the levels of VEGF in MCF-7 cells (**C**) and HUVECs (**D**) treated with CA4 for 12 h at normoxia and then subjected to an additional 12 h of hypoxia. Secretion of VEGF in culture medium measured by ELISA in MCF-7 cells (**E**) and HUVECs (**F**) treated with CA4 (10 nM) at normoxia for 24 h or at normoxia for 12 h and then subjected to an additional 12 h of hypoxia. All data were represented as means ± SD, n = 3, *P < 0.05 versus control, **P < 0.01 versus control, ***P < 0.001 versus control.

To investigate the molecular mechanism of CA4 induced inhibition of angiogenesis, we next investigated the effect of CA4 on VEGF expression levels under normoxic and hypoxic conditions by Western blot. Results in Fig. 5A,B revealed that both in MCF-7 cells and HUVECs, treatment with CA4 in concentrations of 5 and 10 nM induced slightly suppression of the VEGF expression in normoxia (p > 0.05). In contrast, treatment with all given concentration (5, 10, 20 nM) of CA4 as adequately to significantly inhibit the expression of VEGF in hypoxic conditions (Fig. 5C,D). VEGF is induced by hypoxia-inducible factor-1α (HIF-1α), under hypoxic conditions. We also showed that the expression of HIF-1α was suppressed after CA4 treatment in MCF-7 cells and HUVECs. Consistent with the western blot results, the secretion of VEGF was also dramatically decreased after CA4 treatment in MCF-7 cells and HUVECs under hypoxia (Fig. 5E,F). Collectively, these observations suggest that CA4 might inhibit the paracrine as well as autocrine effects of VEGF in tumors and thus exert direct anti-angiogenic effects.

### CA4 suppressed activation of VEGFR-2 and its downstream signaling pathways

**Figure 6:**
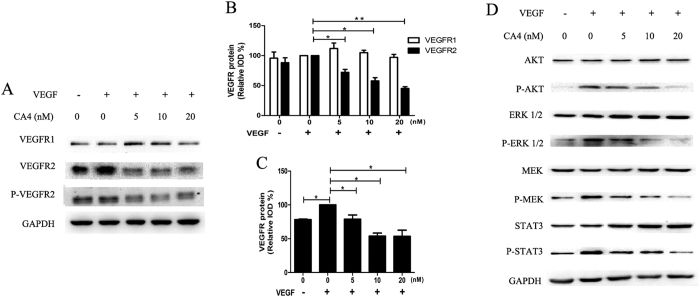
CA4 suppressed VEGFR-2 signaling in HUVECs. A representative Western blot shows the levels of VEGFR1, VEGFR-2 and p-VEGFR-2 in HUVECs treated with CA4 for 24 h following VEGF stimulation (**A**). Histogram of relative VEGFR1 and VEGFR-2 expression levels in HUVECs as determined by western blot analysis (**B**). Histogram of relative p-VEGFR-2 expression levels in HUVECs as determined by western blot analysis (**C**). A representative Western blot shows the levels of total and phosphorylated AKT, ERK, MEK, Stat3 in HUVECs treated with CA4 for 24 h following VEGF stimulation (**D**). All data were represented as means ± SD, n = 3, *P < 0.05 versus control, **P < 0.01 versus control, ***P < 0.001 versus control.

We next examined the protein expression of the VEGF receptors in HUVECs based on western blot analysis. Our data showed that the basilar VEGFR-2 protein expression was down-regulated after CA4 treatment, whereas VEGFR1 protein expression was not discernibly affected (Fig. 6A,B). Treatment with CA4 also decreased VEGF-induced VEGFR-2 phosphorylation in HUVECs. These results suggest that CA4 inhibits VEGFR-2 activity (Fig. 6A,C).

We further investigated the effects of CA4 on downstream mediators of VEGFR-2 in HUVECs. As shown in Fig. 6D, CA4 reduced VEGF-stimulated activation of the downstream moleculars of VEGFR-2 in a concentration-dependent manner in HUVECs, including AKT, ERK 1/2, MEK and Stat3. In contrast, total levels of AKT, ERK 1/2 and MEK were not dramatically affected by CA4 treatment. The above results revealed that CA4 inhibited angiogenesis by directly targeting VEGF stimulated VEGFR-2 activation in endothelial cell, and further suppressed VEGFR-2 downstream signaling pathways.

### CA4 inhibited tumor growth and angiogenesis in a MCF-7 mouse xenograft model

**Figure 7:**
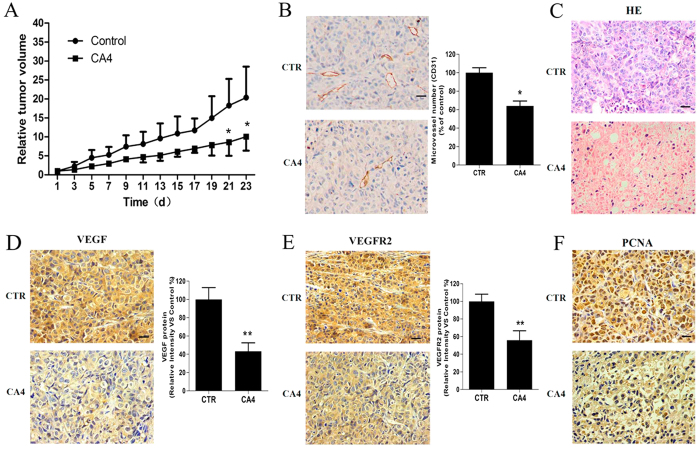
CA4 inhibited tumor growth and neoangiogenesis in MCF-7 breast cancer xenografts model. Tumor volume of animals treated with 15 mg/kg CA4 or vehicle as control daily for 23 d (**A**). (left) Representative image of tumor section stained with CD31; (right) Histogram of microvessel number in tumor sections (**B**). Representative image of hematoxylin–eosin (HE) staining of tumor tissue sections (**C**). (left) Representative image of tumor section stained with VEGF; (right) Histogram of VEGF expression levels in tumor sections (**D**). (left) Representative image of tumor section stained with VAGFR2; (right) Histogram of VEGF expression levels in tumor section (**E**). Representative image of tumor section stained with PCNA (**F**). Images were all 40×, bar = 20 μm. All data were represented as means ± SD, n = 5, *P < 0.05 versus control, **P < 0.01 versus control.

To elucidate the anti-tumor effects of CA4 *in vivo*, the tumor growth in MCF-7 bearing mice following treatment with 15 mg/Kg/day CA4 was investigated. As shown in Fig. 7A, CA4 treatment significantly suppressed the tumor growth. The relative tumor volume of CA4 treated group was 51.6% compared to control group at the end of the 23 d therapy period.

We further investigated the anti-angiogenic activity of CA4 *in vivo* by performing immunohistochemistry with CD31-specific antibody to stain solid tumor sections as CD31 is a widely used endothelial marker for quantifying angiogenesis. As shown in Fig. 7B, the microvessel density (MVD) in CA4 treated tumors was dramatically less than that in control group.

The H&E staining suggested that after CA4 treatment, tumors exhibited large areas of late-apoptotic or necrotic cells (Fig. 7C). Consistent with the *in vitro* results, VEGF and VEGFR-2 protein levels in tumors were reduced in CA4 treatment group when compared with that in control group (Fig. 7D,E). In addition, as shown in Fig. 7F, CA4-treatment significantly reduced cell proliferation in tumors as determined by immunohistochemistry for PCNA, which can be used as an index of cell proliferation.

## Discussion

Angiogenesis is defined as the formation of new blood vessel growth from existent micro vessels and involves several processes, including proliferation of endothelial cells, proteolytic degradation of the extracellular matrix and migration of endothelial cells, leading to the organization of endothelial cells into capillary-like structures. In this study, our results showed that CA4 induced a significant inhibitory effect on endothelial cell proliferation both in the absence and in presence of VEGF. We also revealed that CA4, at nanomole concentration range, could dramatically inhibit VEGF-stimulated migration and capillary-like tubes organization of HUVECs. Nevertheless, the above *in vitro* assays lack the biological complexity of vascular system in vertebrate animals. Supporting evidences concerning *in vivo* anti-angiogenic effects of CA4 then came from *ex vivo* and *in vivo* models. It was found that CA4 significantly blocked microvessels sprouting from rat aortic rings, reduced the microvessel density in CAM model and tumor tissues of MCF-7 xenograft model. In addition, CA4 also induced significant tumor growth inhibition in the mouse model bearing human breast cancer. These data suggest that CA4 might inhibit angiogenesis through inhibition of VEGF-induced endothelial cell functions involved in angiogenesis.

In tumors, cells are initially oxygenated and nourished by simple diffusion depending on pre-existent vasculature, but when tumor tissues grow beyond the limit of oxygen/nutrient diffusion, the tumor microenvironment becomes dominant with hypoxia^19^. For further growth, tumors induce the transcription and secretion of pro-angiogenic factors controlled by hypoxia-inducible factor (HIF) to develop an angiogenic phenotype^20^. Among these endogenous pro-angiogenic factors, the vascular endothelial growth factor (VEGF) appears to play a dominant role^21^,^22^. It is now well-established that VEGF stimulates numerous steps within the tumor angiogenesis, including endothelial cells proliferation and migration^23^. We showed that CA4 suppressed the expression of VEGF both in MCF-7 cells and HUVECs, suggesting that CA4 might inhibit tumor angiogenesis through both paracrine and autocrine VEGF mechanisms.

VEGFR-2 is the predominant mediator of VEGF stimulated angiogenic associated functions of endothelial cell and VEGFR-2 phosphorylation and initiates downstream signaling pathways including ATK/ERK signaling cascade^24^. Activation of the AKT, ERK and MEK has been shown to regulate cell proliferation, differentiation and migration functions^21^,^25^. Phosphorylated Stat3 is reported to regulate cell functions such as migration and proliferation^26^,^27^. In contrast to VEGFR-2, VEGFR1 has weak tyrosine kinase phosphorylation activity^28^. Following stimulation with VEGF, activation of VEGFR1 has no direct effects^24^. In the present study, we demonstrated that VEGF stimulated the proliferation of HUVECs and inhibition of VEGFR-2 by SU1498 significantly inhibited the proliferation of HUVECs. Our data showed that CA4 significantly downregulated the expression level of VEGFR-2, whereas it did not affect VEGFR1 protein expression level. Meanwhile, CA4 significantly inhibited VEGF-stimulated phosphorylation of VEGFR-2 and its downstream molecular including AKT, ERK, MEK and Stat3 in HUVECs, which indicated its ability to block angiogenesis.

Taken together, our data suggest that the mechanism by which CA4 exerts its anti-angiogenic effect is at least in part through suppression of the VEGF/VEGFR-2 signaling. This clarification may likely help to better define the therapeutic potential and clinical indications of CA4 in treating tumor.

## Materials and Methods

The animal experiment was approved and conducted according to the regulations set by the Animal Use and Care Committee of Central South University. All experiments were carried out in accordance with the manufacturer’s instructions.

### Materials

Medium M199, Dulbecco’s modified Eagle’s medium (DMEM), RPMI (Roswell Park Memorial Institute)-1640 medium, fetal bovine serum (FBS) were obtained from Hyclone Laboratories (South Logan, utah, USA). VEGF and SU1498 were ordered from PeproTech (Rocky Hill, NJ, USA) and Calbiochem (Merck KGaA, Darmstadt, Germany). Matrigel were obtained from BD Bioscience (Bedford, MA, USA). All antibodies were ordered from Cell Signaling Technology (Danvers, MA, USA) or Abcam (Louis Park, MN, USA). CA4 was synthesized as described^14^,^29^. For the *in vitro* studies, CA4 was dissolved in dimethyl sulfoxide (DMSO as vehicle) at a concentration of 10 mM and then subsequently diluted in culture medium.

### Cell culture

HUVECs and MCF-7 cells were grown in M199 and DMEM medium supplemented with 10% FBS, respectively. T47D, A549, SW480 and 5–8 F Cells were grown in RPMI-1640 medium supplemented with 10% FBS. Cells were all incubated at 37 °C in a 5% CO2 atmosphere. All cells were cultured 24 hours before incubated with corresponding treatments.

### Cell proliferation assay

Cell proliferation was analyzed using the 3-(4, 5-dimethyl-2-thiazolyl)-2, 5-diphenyl-2H-tetrazolium bromide (MTT) assay. Cells were seeded in 96-well plate, after treatment with vehicle or serial concentration (0, 1, 5, 10, 50, 100 nM) CA4 for 48 h, 20 μL MTT (2 mg/mL, Sigma-Aldrich, Saint Louis, MO) was added to each well for 3 h. The medium was removed and the Formazan in each well were dissolved in 100 μL DMSO. The optical density (OD) of the wells was measured on a microplate reader at 490 nm. In addition, the HUVECs proliferation in the presence of VEGF (20 ng/mL) or SU1498 (10 μM) were also measured.

### Lactate dehydrogenase (LDH) toxicity assay

The LDH release is an index of cytotoxicity and evaluation of the permeability of cell membrane^30^. Cells were seeded in 96-well plate. After incubation with vehicle or various concentrations of CA4 for 48 h, cell supernatants were collected and analyzed using LDH cytotoxicity assay kit from Beyotime (Shanghai, China). The absorbance of formed formazan was read at 490 nm on a microplate reader.

### Cell cycle analysis

HUVECs were seeded in 6-well plate. After incubated with vehicle or CA4 for 24 h, both floating and adhesive cells were collected and fixed with ice-cold 75% ethanol overnight at 4 °C. The cells were then stained with a cell cycle analysis kit (Beyotime, Shanghai, China) at room temperature in the dark for 30 min. Cell cycle distribution was determined via FACScan flow cytometer (BD FACS Calibur).

### Wound-scratch migration assay

HUVECs were allowed to form a confluent monolayer in 6-well plate. The cells were subsequently wounded with 10 μL pipette tips and washed triple with phosphate-buffered saline. M199 medium with 1% FBS and VEGF (20 ng/mL) containing vehicle or CA4 was then added to the wells. At indicated time points, the wounds were photographed using a light microscope. The percent of gap closure was calculated by measuring the wound width. The migration rate was calculated by the formula: (width_0h_–width_24h_)/width_0h_ × 100%.

### *In vitro* formation of capillary-like tube structures

70 μl Matrigel was added to 96-well plates and then allowed to polymerize at 37 °C for 1 h. HUVECs were carefully seeded on top of the polymerized Matrigel, treated with VEGF (20 ng/mL) and vehicle or CA4. After incubation for 2 h, tube formation were observed and photographed under a light microscope. The effect of CA4 on tube formation was calculated by measuring the length of capillary-like network.

### Aortic ring assay

Rat aortic ring assay was performed as described in previous studies^31^. Aortic rings harvested from male Sprague-Dawley rats were sectioned into 1 mm-long cross sections, placed on the surface of 100 μl Matrigel in 48-well plates and covered with an additional 60 μl Matrigel. The rings were cultured in 300 μl growth medium which contained M199 medium with 20% FBS and either vehicle or CA4. The aortic rings were cultured in CO_2_ incubator at 37 °C and the medium was replaced every 48 h. The microvessel growth was photographed after cultured for 6 days. The microvessel sprouting was quantified by counting the number of microvessels from the rat aortic rings.

### Chicken chorioallantoic membrane (CAM) assay

Anti-angiogenic activity of CA4 on CAM was assayed as described previously^32^. Groups of 10 fertilized chicken eggs were transferred to an egg incubator, incubated at 37.8 °C and 60–70% relative humidity for 8 d. After this incubation, a small hole was drilled on the broad end of the egg and a window was carefully created on the eggshell. Sterilized filter paper disks (5 × 5 mm) saturated with either vehicle or CA4 was placed on the CAM. The windows were then sealed with cellophane tape. After incubated for another 2 d, the CAMs were photographed. Angiogenesis was quantified by manually counting the number of blood vessel branch points.

### Western blot analysis

After treatment, cells were collected and lysed in lysis buffer (RIPA buffer containing a proteinase inhibitor and phosphatase inhibitor (Sigma-Aldrich, St. Louis, MO)). Cell lysates were clarified by centrifugation at 15,000 g for 20 min at 4 °C. The equal amounts of lysates were separated by 10% sodium dodecyl sulfate-polyacrylamide gel electrophoresis (SDS-PAGE) and transferred to polyvinylidene difluoride (PVDF) membranes. Membranes were then blocked and incubated with specific antibodies overnight at 4 °C followed by incubated with secondary antibodies 1.5 h at 37 °C. Immunolabeling was detected using an enhanced chemiluminescence (ECL) detection system according to the manufacturer’s instructions. Protein expression was calculated using Quantity One software 4.1.0.

### VEGF ELISA assay

VEGF secretion was measured in MCF-7 cells and HUVECs using a VEGF immunoassay kit (R&D Systems, Inc., inneapolis, MN, USA). Cells were incubated with vehicle or CA4 (10 nM) for 24 h under indicated conditions. Supernatants were collected and the levels of VEGF protein were measured according to the manufacturer’s protocol.

### *In viv*o human breast cancer xenografts

Female BALB/c nude mice, weighing 18–22 g, were obtained from the Experimental Animal Center of Central South University (Changsha, China). The mice were kept in an environmentally controlled breeding room under 12 hours’ light-dark cycles with controlled temperature and humidity. 1 × 10^7^ MCF-7 cells were injected subcutaneously in the right flank of the mice. After the tumors’ growth to about 100 mm^3^, mice were randomly divided into two groups (n = 5) and treated intraperitoneally with vehicle or CA4 (15 mg/kg/day). The body weight and tumor size of each mouse were record every other day. The tumor volume (V) was determined using digital vernier caliper measurements and calculated as V = (A × B^2^)/2, where A is the longest diameter while B is the shortest diameter of the tumor.

### Immunohistochemistry analysis

After treatment for 23 days, the mice were sacrificed under anesthesia. Tumors were harvested, fixed in 10% buffered formalin solution, embedded in paraffin and sectioned (thickness of 6 μm). Histological assessment was thus made by staining with hematoxylin and eosin (H&E) to assess the induction of Necrosis. The tumor sections were immuno-stained with antibodies including CD31, PCNA, VEGF and VEGFR-2 and examined with light microscopy.

### Statistical analysis

All data are expressed as mean ± Standard deviation (SD). In this study, Statistical analysis was conducted using SPSS 18.0 software (SPSS, Chicago, IL, USA). Quantitative data were analyzed by using one-way ANOVA. The difference was considered to be significant at p < 0.05.

## Additional Information

**How to cite this article**: Su, M. *et al*. The anti-angiogenic effect and novel mechanisms of action of Combretastatin A-4. *Sci. Rep.* **6**, 28139; doi: 10.1038/srep28139 (2016).
